# Extracellular microRNAs as messengers in the central and peripheral nervous system

**DOI:** 10.1042/NS20170112

**Published:** 2017-11-02

**Authors:** Hannah Scott

**Affiliations:** School of Pharmacy and Pharmaceutical Sciences, Cardiff University, Cardiff CF10 3NB, U.K.

**Keywords:** central nervous system, gene expression and regulation, intracellular signaling, microRNA, microparticles, trafficking

## Abstract

MicroRNAs are small post-transcriptional regulators that play an important role in nervous system development, function and disease. More recently, microRNAs have been detected extracellularly and circulating in blood and other body fluids, where they are protected from degradation by encapsulation in vesicles, such as exosomes, or by association with proteins. These microRNAs are thought to be released from cells selectively through active processes and taken up by specific target cells within the same or in remote tissues where they are able to exert their repressive function. These characteristics make extracellular microRNAs ideal candidates for intercellular communication over short and long distances. This review aims to explore the potential mechanisms underlying microRNA communication within the nervous system and between the nervous system and other tissues. The suggested roles of extracellular microRNAs in the healthy and the diseased nervous system will be reviewed.

## Introduction

MicroRNAs, short non-coding RNAs, have emerged as powerful regulators of gene expression. MicroRNAs in their mature processed form are single-stranded RNA molecules, 18–24 nucleotides in length. These post-transcriptional regulators bind to complementary or near-complementary sequences in 3′-untranslated regions (3′-UTR) of mRNA molecules, leading to degradation of the transcript or translational repression respectively [1]. In the central nervous system (CNS), microRNAs have been shown to be important for a wide range of physiological and pathophysiological processes. MicroRNAs are thought to play a role in neuronal function and have also been found to be dysregulated in a range of neurological diseases [2–4].

As new microRNAs are discovered, interest in these small RNAs as diverse regulators of gene expression has steadily increased, in particular because microRNAs are ubiquitously expressed, yet display tissue-specific expression profiles [5]. It has been estimated that approximately two-thirds of human protein-coding genes are regulated by microRNAs [6]. In fact, each mRNA is thought to be regulated by multiple different microRNAs [6]. Conversely, each microRNA has been shown to regulate a wide range of different mRNA targets [7]. Therefore, gene regulation by microRNAs represents a complex network that may fine-tune gene expression in a dosage-dependent manner [8].

The recent discovery that microRNAs can be found extracellularly, as a potential novel type of intercellular messenger [9–12], has added to the complexity of microRNA regulation. MicroRNAs have been detected circulating in blood and cerebrospinal fluid (CSF), as well as in most other body fluids [13]. Intriguingly, while RNA molecules in the cell tend to be short-lived, circulating microRNAs show much higher stability and are protected from degradation by endogenous RNases [14]. Altered levels of specific microRNAs in body fluids have been linked to a wide range of diseases, including disorders of the nervous system [15–28]. Serum microRNA levels were altered in children with autism spectrum disorders and salivary levels have been correlated with neurodevelopmental scores [15,16]. CSF, plasma or serum microRNAs abnormalities were associated with a wide range of neurological diseases including Alzheimer’s disease [17–20], Parkinson’s disease [16,21], Huntington’s disease [22] and multiple sclerosis [23,24], as well as glioma [25–27] and stroke [28]. MicroRNA levels in the CSF were used to differentiate between glioblastoma and brain metastatic cancers [27]. Patients with poor outcomes following acute ischaemic stroke were associated with greater levels of circulating miR-223 [28]. In peripheral blood plasma of multiple sclerosis patients, microRNA levels were correlated with severity of disease and frequency of flare-ups [24]. Furthermore, age-related changes in circulating microRNA levels have been noted in mouse and human [29,30]. Based on these studies, circulating microRNAs may be used as biomarkers for disease diagnosis and likelihood of remission or treatment success.

Intriguingly, extracellular microRNAs may be more than just markers that are released by diseased tissue. They are actively secreted by healthy cells, including cells of the nervous system [31–35], and can be taken up by neighbouring cells or even other tissues, where they are functionally active [31,36–41]. Hence, extracellular microRNAs have the potential to act as messengers over both short and long distances [9–11]. This review aims to summarize in what form microRNAs have been found extracellularly, the export and uptake mechanisms of microRNAs within exosomes and the potential role these extracellular microRNAs may play in nervous system function and disease.

## MicroRNAs are found extracellularly in a variety of forms

**Figure 1:**
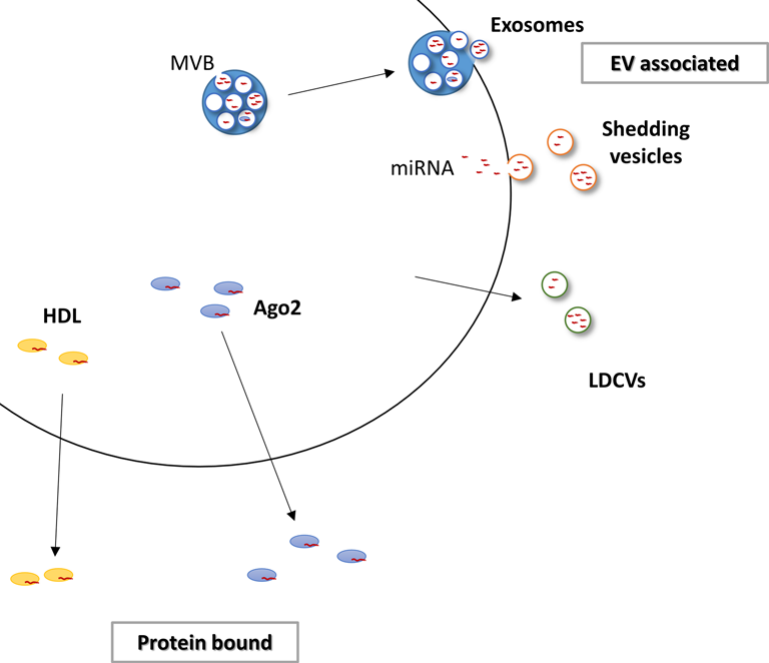
Methods of microRNA release Extracellular microRNAs can be secreted in extracellular vesicles (EV), in the form of exosomes, derived from inward budding of the multivesicular body (MVB) membrane and docking of the MVB with the plasma membrane; within shedding vesicles, formed by outward budding of the plasma membrane; or in large dense-core vesicles (LDCVs). MicroRNA can also be bound to proteins, in particular to argonaute 2 (Ago2) or high-density lipoproteins (HDL).

The increased stability of extracellular microRNAs is due to the fact that they are protected from degradation by being packaged into extracellular vesicles (EVs) [9,31,32,36–39,42–45] or bound to proteins [46–50] (Figure 1). A variety of different vesicles are actively secreted from cells. MicroRNAs have been detected in exosomes [9,31,36–39,42–44], shedding vesicles [32] and large dense-core vesicles (LDCVs) [45]. Proteins that have been found associated with extracellular microRNAs are argonaute 2 (AGO2) [46–48], a component of the microRNA processing machinery, and high-density lipoproteins (HDL proteins) [50].

### Extracellular vesicles

#### Exosomes

The most well-characterized form of EVs is exosomes. Thirty to 100 nm in size, they are small vesicles made up of a lipid bilayer. They are most commonly isolated using ultracentrifugation and concentrated by sucrose gradient centrifugation where they float with a buoyant density of 1.10–1.19 g/ml. The exosome cargo has been shown to consist of lipids, proteins and different RNA species. Exosomes contain mRNA and small RNAs including microRNA, but no or little ribosomal RNA [9,37–39]. Differences between sequences of exosomal microRNAs and genomic sequences suggested that microRNAs in exosomes may have been edited and trimmed [42]. Analysis of microRNAs isolated from exosomes indicated that most mapped to the mature targeting strand, which suggests a potential functional effect [43]. Additionally, components of the RNA processing machinery, including Dicer, AGO2 and TRBP have been detected in some exosomes [32,40]. However, the possibility of microRNA processing within exosomes has not been explored. Analyses of the relative proportion of microRNAs amongst total small RNAs within exosomes have produced varied results, possibly depending on producer cell type, physiological conditions and analysis method. Relative levels ranging from 3% in neural stem cell-derived exosomes up to 20% in cancer cell line-derived exosomes have been reported [31,42,44]. The consensus suggests however that the proportion of microRNAs in exosomes tends to be much lower than within the cell [31,42,44].

Exosomes are characterized and distinguished from other vesicle types, by their origin within the cell. Exosomal vesicles are formed within late endosomal structures called multivesicular bodies (MVB) by inward budding of the membrane, using the endosomal sorting complex required for transport (ESCRT) machinery [51]. Alternatively, in an ESCRT-independent pathway, formation of these intraluminal vesicles requires ceramide, which is produced from sphingomyelin by neutral sphingomyelinase 2 (nSMase2) [52]. MVBs fuse with the plasma membrane to release their exosome cargo into the extracellular space. In addition to carrying membrane proteins specific to their origin cell, exosomes are enriched in a range of exosomal markers. These include tetraspanins CD9, CD63 and CD81, heat-shock proteins HSP70 and HSP90, as well as ALIX and TSG101, which are components of the ESCRT machinery. However, use of these proteins as exosomal markers has become controversial as several have been detected on non-exosomal EVs and some may only be presented on certain subtypes of exosomes [53].

MVBs have been identified in neurons, most commonly in post-synaptic densities, and within the major types of glial cell [54], including oligodendrocytes, where MVBs were predominantly found in the adaxonal loop, the innermost wrapping closest to the axon [55]. A range of cell types of the nervous system have been shown to release exosomes. Exosomes are secreted from rat cortical neurons in culture [56], as well as from astrocytes [57], oligodendrocytes [55], microglia [58] and human neural stem cells [36]. Along with traditional exosomal markers ALIX and Tsg101, neuron-derived exosomes also contained neuron-specific cell-adhesion protein L1 [56]. Exosomes derived from cultured neurons or neural stem cells contained microRNAs, including miR-124a, one of the most abundant microRNAs in the CNS [31]. MicroRNA-carrying exosomes can be taken up by cells in the nervous system and exert their function there. Exosomes produced by neurons have been shown to be internalized by astrocytes and transfer miR-124a, leading to altered expression of downstream targets [31]. Neural stem cell-derived exosomes carrying miR-1246 successfully down-regulated its target in HeLa cells as assessed by luciferase assay [36]. Exosomes isolated from oligodendrocyte-conditioned medium were taken up predominantly by microglia and neurons while only a small proportion of astrocytes and oligodendrocytes internalized these exosomes [55]. These results suggest a target cell specificity of nervous system exosomes.

#### Shedding vesicles

Shedding vesicles are generally distinguished from exosomes by their larger size of 100–200 nm. Distinctly, shedding vesicles are released by direct outward budding of the plasma membrane. Certain plasma membrane marker proteins have been associated with these vesicles, such as β1 integrin [59]. Shedding of vesicles is dependent on activation of ATP receptor P2X7 [60] and has been observed from microglia [60], astrocytes [61] and neurons [62]. MicroRNAs along with AGO2 have been detected within shedding vesicles isolated from astrocytes. These vesicles were internalized by neuroblastoma cell line SH-SY5Y and primary neurons where the transported microRNAs were functionally active [32].

#### Large dense-core vesicles

Recently a third class of EVs, LDCVs of 100–200 nm size, has been associated with active microRNA export from cells [45]. The study by Gümürdü et al. [45] detected RNA within these large vesicles of which over 60% constituted microRNAs and which contained no ribosomal RNA. Interestingly, LDCVs, which are also known to carry neuropeptides, hormones and amines, utilize the process responsible for releasing synaptic vesicles from neuronal cells [63] and may therefore be of particular interest as a vehicle for microRNA release in the nervous system. So far only one study has measured microRNAs in LDCVs. It is clear that more work needs to be done to characterize microRNA-containing LDCVs from other tissue types and investigate if they are taken up by recipient cells and retain their repressive function.

### Proteins

While evidence points towards a role for EVs in the intercellular transport of microRNAs, multiple studies have found that only a minority of total extracellular microRNAs, as low as 2.5%, were associated with EVs [46,47,49,64,65]. The proportion of microRNAs associated with EVs may be specific to the individual microRNA. Different populations of extracellular microRNAs have been detected in plasma and conditioned medium that were either enriched in EVs or preferentially associated with proteins or found in both [46,49].

AGO2 is an RNA-binding protein and the catalytic component of the RNA-induced silencing complex (RISC). AGO2 binds microRNAs which guide the protein to the target mRNA, leading to cleavage of the mRNA by the PIWI domain or translational inhibition through recruitment of other gene-silencing proteins [66,67]. While AGO2 has been detected within EVs [32,40], in HEK293T cell-conditioned medium the large majority of extracellular AGO2 was not associated with vesicles [43]. Other studies have detected extracellular microRNAs bound to AGO2 and possibly to other Argonaute family members [46,47]. In synaptosomes, preparations of sealed functional presynaptic nerve terminals, a proportion of microRNAs were found to be released associated with AGO2 [48]. It does not seem unreasonable that extracellular microRNAs may also be bound to other types of RNA-binding proteins. In fact, nucleolar RNA-binding protein, NPM1, may also bind extracellular microRNAs and protect them from degradation by RNases [49].

Furthermore, HDL proteins isolated from human plasma were found to be associated with microRNAs, but not mRNAs, and were taken up by recipient cells where the microRNAs were functionally active [50]. Further characterization of proteins that carry extracellular microRNAs is required to gain a better understanding of the breadth of microRNAs being released from cells.

### Physiological significance of extracellular microRNAs

A recent study queried whether microRNA content in exosomes is of physiological significance. Stoichiometric analysis of microRNA content within exosomes isolated from different cells and fluids showed on average less than one microRNA per exosome [64]. Another study measured higher copy numbers of individual microRNAs, which they considered sufficient to repress mRNA targets [31]. Chevillet et al. [64] suggested the possibility of a low-occupancy/high-microRNA model, where different exosomal subpopulations exist, some of which are rich in certain microRNAs, which are taken up selectively by recipient cells. In fact, subpopulations of exosomes have been identified from different cell sources and these may carry specific cargo [53,68–70]. In contrast to exosomes, each LDCV, isolated from bovine adrenal medulla chromaffin cells, seemed to contain numerous copies of microRNAs. The most abundant microRNA miR-375 was quantified at 400–500 copies per vesicle [45]. While the majority of extracellular microRNAs are reportedly not associated with EVs, it is not known how efficiently protein-bound microRNAs are taken up by recipient cells. Functionally however, it has been demonstrated in multiple studies that extracellular microRNAs can be taken up by other cells and down-regulate target mRNAs in the recipient cells, leading to physiological effects [37–41].

## Export and uptake mechanisms of extracellular microRNAs

Efforts have been made to understand the mechanisms that govern microRNA release in its various forms from the cell, in general as well as in the nervous system. Most of the research investigating release and uptake mechanisms of vehicles carrying microRNAs has concentrated on exosomes. Therefore, this section will focus on the current literature regarding exosomal mechanisms. Relatively little is known about mechanisms underlying protein-bound microRNA export and uptake. Briefly, apart from being secreted from synaptosomes, AGO2-bound microRNAs were also detected within the synaptosomes where approximately one-third were encapsulated in synaptic vesicles [48]. Similar to secretion of neurotransmitters at the synapse, microRNA secretion from the synaptosomes was enhanced by depolarization and may occur by Ca^2+^-dependent exocytosis [48]. This raises the possibility that AGO2-bound microRNAs may be stored within synaptic vesicles alongside neurotransmitters in neurons although currently there is little evidence of this process.

### Export mechanisms

#### MicroRNA loading into exosomes

**Figure 2:**
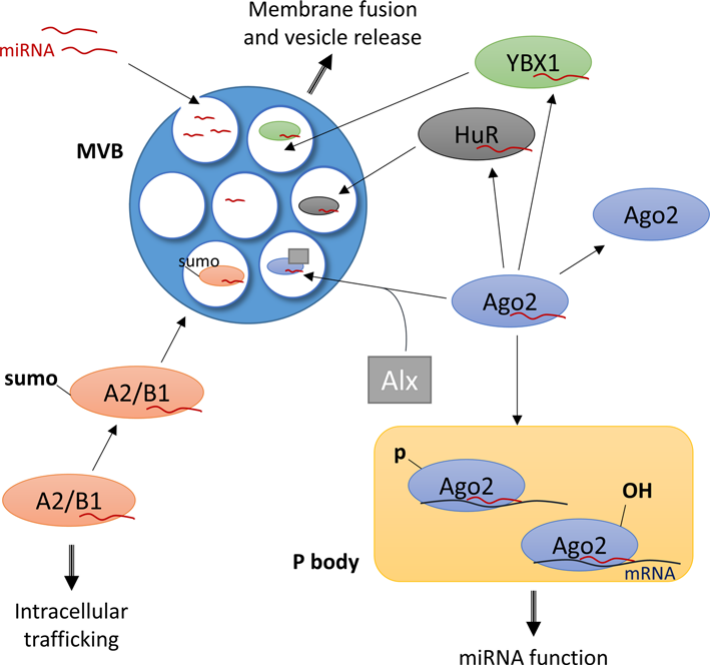
Uptake of microRNAs into exosomes MicroRNAs are taken up into exosomal vesicles when they bud inwards from the multivesicular body (MVB) membrane. Several proteins have been identified that promote incorporation of microRNAs into exosomes. Argonaute 2 (Ago2) binds microRNAs and is found within exosomal vesicles, supported by Alix (Alx) which interacts with Ago2 and increases Ago2 localization into extracellular vesicles. In contrast, phosphorylated or hydroxylated Ago2 is mainly found within P bodies, where it supports microRNA function on mRNA targets. HuR and Y-box binding protein 1 (YBX1) uncouple microRNAs from Ago2 and bind the microRNA themselves which promotes uptake into exosomes. Heterogeneous nuclear ribonucleoprotein A2/B1 binds and transports RNA molecules in neurons. Sumoylated A2/B1 is preferentially loaded into exosomes.

The observation that relative levels of microRNAs in EVs differ from their levels in the cell of origin, together with the finding that some microRNAs are preferentially released while others are preferentially retained in the cell [31,42–44,71], suggests that some microRNAs are selectively packaged into exosomes and secreted. Several microRNA-interacting proteins have been identified that regulate whether intracellular activity of microRNAs or their loading into exosomes is favoured in the cell (Figure 2).

AGO2 deletion in a series of different cell types reduced the EV-mediated release of a number of highly exported microRNAs [71]. Therefore, AGO2 may play an important role in the export of these particular microRNAs. Evidence comes from studies that characterized the effects of AGO2 post-transcriptional modification on protein function [65]. AGO2 that was hydroxylated at Pro-700 in its PIWI domain, which is responsible for the cleavage of target RNA molecules, increased the target repression by bound microRNA miR-210, while non-hydroxylated AGO2 promoted the cellular release of miR-210 [65]. Whether AGO2-bound microRNAs are preferentially released within EVs or in their soluble protein-bound form was not investigated in this study. Similarly, phosphorylation of AGO2 at Ser-387 by MEK/ERK signalling increased localization of AGO2 in so-called processing bodies (P bodies), which are hubs for microRNA function in the cell. Inhibition of AGO2 phosphorylation shifted AGO2 localization to the MVB and increased its association with exosomes [72]. While it was not investigated in this study, it is possible that AGO2 within MVBs is associated with microRNAs. These findings point towards AGO2 modification as a switch for microRNA localization in the cell. ESCRT family member ALG 2-interacting protein X (ALIX) may associate with AGO2 and promote its incorporation into EVs, as knockdown of ALIX was found to reduce AGO2 and microRNA compartmentalization in EVs [73].

A few other RNA-binding proteins have been implicated in regulating microRNA loading into exosomes. Y-box binding protein 1 (YBX1), which regulates translation in response to neuronal activity [74], and human antigen R (HuR), an important regulator of neocortex development [75], were detected within exosomes in HEK293T cells and hepatic cells respectively. Both proteins reduced microRNA function by dissociating microRNAs from AGO2 and increased exosomal levels of these microRNAs [43,76]. Ubiquitination of HuR within the MBV caused uncoupling from microRNAs and their export within exosomes [76]. Heterogeneous nuclear ribonucleoprotein A2/B1 (hsRNPA2/B1) is known to bind to an RNA trafficking sequence and transport mRNAs to dendrites [77]. This RNA-binding protein has also been shown to associate with specific microRNA sequences, termed EXOmotifs, and promote exosomal loading of these microRNAs. Sumolyation of hnRNPA2/B1 may provide the switch between intracellular trafficking and sorting into exosomes [78]. Due to its known role in neurons, this protein may be of particular importance for microRNA export in the nervous system. Other proteins that have been implicated in the recruitment of microRNAs into EVs or in the selective export of microRNAs are oncogene KRAS [42], membrane-binding protein Annexin A2 [79], transcription factor KLF2 [80] and Rab family proteins [80]. However, their exact mechanisms are unknown.

Specific sequence motifs have been identified that may guide the selective packaging of certain microRNAs [78,81]. Villarroya-Beltri et al. identified two EXOmotifs, GGAG and CCCU, which are over-represented in microRNAs localized within exosomes. Converting an EXOmotif into a motif present in predominantly cellular microRNAs, or vice versa, altered microRNA localization [78]. A further study confirmed an enrichment of G-rich sequences amongst exosomal microRNAs [81]. Post-transcriptional modifications may also play a role in exosomal targeting of microRNAs. 3′-end-uridylated microRNAs were preferentially detected in exosomes while 3′-end-adenylation was enriched amongst cellular microRNAs [44]. How these modifications are detected and lead to differential distribution of the microRNAs has not been explored. It is likely that RNA-binding proteins may play a role in regulating microRNA loading into EVs via EXOmotifs. Janas et al. proposed that the above-mentioned motifs and modifications may permit selective binding of the microRNA to lipid raft-like regions of the MVB limiting membrane, as these regions are undergoing inward budding [82]. This hypothesis remains to be tested experimentally.

It has also been suggested that microRNA sorting into exosomes may depend on the amount of mRNA targets present in the cell [83]. Further work is needed to establish the regulatory mechanisms behind selective microRNA export and specifically its role in the nervous system.

#### Secretion of exosomes

Exosomes are released from cells following fusion of MVBs with the plasma membrane. An increase in intracellular Ca^2+^ up-regulated exosome release in an erythroleukemia cell line [84]. Due to the role of Ca^2+^ in neuronal signalling, this may be a relevant mechanism for exosomal secretion regulation in the nervous system. In fact, exosome secretion from cultured neurons isolated from cortex or hippocampus was found to be regulated by Ca^2+^ influx and glutamatergic activation [85]. Moreover, induction as well as inhibition of neuronal activity have been shown to increase release of exosomes and exosomal microRNAs [31,56,85,86]. Exosome release from oligodendrocytes or microglial cells was induced by activation of glutamatergic (AMPA or NMDA) and serotonergic receptors respectively, in a Ca^2+^-dependent manner [55,85,87,88]. The involvement of neuronal activity and neurotransmitters in the release of exosomes strongly supports the hypothesis that exosomes may play a role in nervous system function. Rab family proteins, such as Rab11, Rab27a/b and Rab35, as well as associated proteins have been identified in different cell types, including in oligodendrocytes, as potential positive regulators of exosome release, by promoting MVB docking or tethering to the cell membrane [55,89–91].

### Uptake mechanisms

Exosomes are taken up by a variety of cells. Some studies have indicated a cell-type specific uptake in the nervous system [31,92]. Little is known about how selective uptake of exosomes is regulated. The composition of tetraspanin complexes has been suggested as a potential contributing factor, specifically the interaction between tetraspanin and specific integrin chains was thought to confer target cell specificity [93,94]. Gap junction protein connexin 43 has also been proposed as a mediator of exosomal uptake into cells, when it is present on both cellular and exosomal membranes [95].

**Figure 3:**
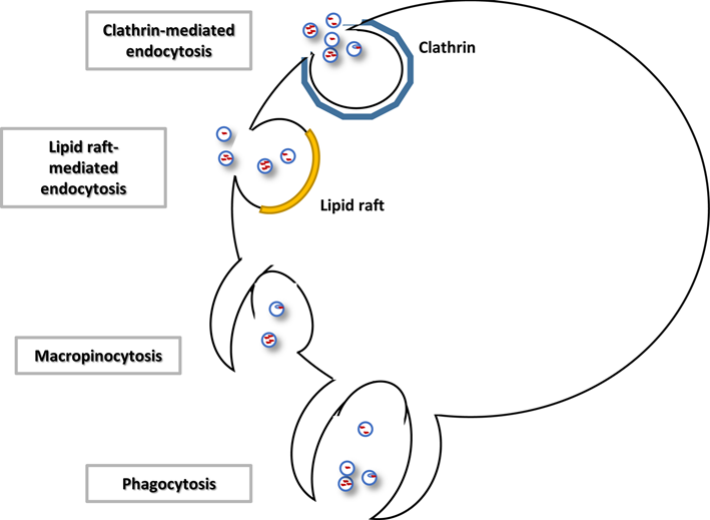
Mechanisms of extracellular vesicle uptake Extracellular vesicles have been shown to be taken up clathrin or lipid raft-mediated endocytosis. Macropinocytosis and phagocytosis have also been observed as uptake mechanisms.

Multiple mechanisms of exosomal uptake have been identified, in some cases within the same cell type (Figure 3). In neurons, clathrin-mediated endocytosis is a predominant mechanism for recycling of glutamate receptors from the synapse [96]. This process has been observed as an uptake mechanism for exosomes in cancer cell lines [97,98], cultured neurons [55] and synaptosomes [48]. Uptake of exosomes by a glioblastoma cell line occurred via lipid raft-mediated endocytosis [99]. Imaging showed filopodia, highly dynamic protrusions of the cell and hotspots for endocytosis, attracting EV localization and supporting their uptake into the cell by surfing, grabbing and pulling within minutes of EV addition [100]. Exosomes incorporated via endocytosis are detected within endosomes [97,99–101] and thought to be transferred to the endoplasmatic reticulum, a centre for translation and mRNA silencing by microRNAs, where they may release their cargo [100]. In synaptosome preparations, internalized microRNAs were recruited to RISC where they were functionally active [48]. Alternatively, internalized exosomes may be targeted to the lysosome for degradation [97,100]. Uptake via invagination of the plasma membrane, or macropinocytosis, has been observed in microglia [102] and a cancer cell line [98]. In dendritic cells, exosomes were observed to partially fuse with the plasma membrane and ‘inject’ their cargo into the cytosol [38]. Lastly, phagocytic cells were able to internalize exosomes via phagocytosis [103].

MicroRNAs that are not packaged into EVs can still be taken up by recipient cells [65]. While extracellular AGO2 has been shown to be transported into cells [65], it is not clear if the AGO2-microRNA association facilitates microRNA uptake. So far only neuropilin 1 (NRP1), a cell surface protein, has been identified as a possible mediator of microRNA uptake. NRP1 binds microRNAs with high affinity, possibly via its b1/b2 domain, and was observed to translocate into the cytoplasm with its RNA cargo [104].

## The roles of extracellular microRNAs in the nervous system in health and disease

As described above, extracellular microRNAs have been shown to be specifically packaged, to be selectively taken up by target cells where they are able to exert their function and to be regulated by cellular processes, including neuronal activity. These characteristics support the hypothesis that extracellular microRNAs act as intercellular messengers and regulators [9–11]. Recent studies have also begun to elucidate the functional contribution of extracellular microRNAs to nervous system health and disease.

### Contribution of extracellular microRNAs to nervous system function

Within the cell, microRNAs have been implicated in diverse biological processes. Nervous system development, such as neuron and glia differentiation [105,106] and axonal growth [107], as well as cognitive processes, including the plasticity mechanisms that govern learning and memory [108], are known to involve post-transcriptional regulation by microRNAs. MicroRNAs support these key processes by targeting a variety of cellular pathways. In neurodevelopment, microRNAs regulate the transition from non-neuronal to differentiated phenotype by targeting transcription factors and splicing factors that promote the expression of neuronal genes [109,110]. Furthermore, localization of microRNAs to specific neuronal compartments enables fast and localized control of mRNA translation [111,112]. A prominent example of local regulation by microRNAs is the regulation of factors involved in the reorganization of the cytoskeleton – in distal axons for the control of axon growth and branching during neural development [113,114] or in dendrites for the regulation of dendritic spine remodelling important for synaptic plasticity [115,116]. MicroRNAs have also been shown to contribute to learning and memory by regulating key plasticity genes, such as CREB1 [117]. Glutamate receptor recycling at the post-synaptic membrane plays a role in modulating synaptic strength thereby contributing to synaptic plasticity [118]. Expression of glutamate receptors and factors required for AMPA receptor exocytosis has been shown to be regulated by a range of microRNAs (reviewed in [119]). Lastly, microRNAs can also modulate nervous system function by targeting cell metabolism through regulation of mitochondrial proteins involved in the respiratory chain [120,121] or in mitochondrial dynamics [122].

Given the involvement of microRNAs in a range of biological processes, extracellular microRNAs that are transported between cells of the nervous system may also contribute to the mechanisms underlying nervous system function. Studies have reported uptake of extracellular microRNAs, potentially within exosomes, by neurons and functional effects of those microRNAs within the recipient cell (Table 1). MicroRNA let-7b induced action potentials in dorsal root ganglion neurons and caused pain-like behaviour in mice [123]. miR-193a within exosomes derived from a differentiated neuronal cell line induced neurogenesis in undifferentiated recipient cells [33]. Exosomal let-7c and miR-21 isolated from neuronal-conditioned medium were internalized by neurons where they activated toll-like receptor 7, a receptor located in the endosome compartment, and regulated dendritic outgrowth [34].

**Table 1:** Known functions of extracellular microRNAs in the nervous system

miRNA	Origin cell	Recipient cell	Vehicle	Role
let-7b [103]	DRG neurons	DRG neurons	Not described	Induction of action potentials via TLR7, nociception
let-7c [32]	Neurons	Neurons	Exosomes	Dendritic growth via TLR7
miR-1 [114]	Glioblastoma cells	Glioblastoma cells	EVs	Tumour suppressive
miR-19a [110]	Astrocytes	Breast cancer cells	Exosomes	Brain metastasis via PTEN
miR-21 [32,111,120]	Neurons, glioblastoma cells, macrophages	Neurons, microglia, macrophages	Exosomes, EVs	Dendritic growth via TLR7; Microglia phenotype, immune response and glioblastoma invasion; Neuronal necroptosis via TLR7
miR-29b [119]	Astrocytes	Neurons	Exosomes	Neuronal survival
miR-34a [30]	Astrocytes	Dopaminergic neurons	Shedding vesicles	Neuron vulnerability, 6-OHDA-induced disease onset
miR-122 [109]	Breast cancer cells	Astrocytes	EVs	Glucose consumption, brain metastasis
miR-124 [29]	Neurons	Astrocytes	Exosomes	GLT1 expression
miR-126 [132]	Brain endothelial cells	Cardiomyocytes	Exosomes	Cardiac health following stroke
miR-132 [33]	Neurons	Brain endothelial cells	Exosomes	Brain vascular integrity
miR-133b [121–124]	Mesenchymal stem cells	Astrocytes, neurons	Exosomes	Promotes exosome release from astrocytes, neurite remodelling, angiogenesis, anti-inflammatory effects, recovery of sensorimotor function and spatial learning
miR-193a [31]	Neurons	Neural progenitor cells	Exosomes	Neurogenesis
miR-451 [111]	Glioblastoma cells	Microglia	EVs	Regulation of microglia phenotype, immune response and glioblastoma invasion

Abbreviations: 6-OHDA, 6-hydroxydopamine; DRG, dorsal root ganglion; EV, extracellular vesicle; GLT1, glutamate transporter 1; PTEN, phosphatase and tensin homolog; TLR7, toll-like receptor 7.

Neuron-derived exosomes can also be taken up by other cell types in the nervous system. Exosomes secreted from neuronal cells were found to bind to neurons, astrocytes and oligodendrocytes but predominantly they were taken up by glial cells [31,92]. Exosomes released from cortical neurons following synaptic activation were internalized by neurons and some were detected bound to pre-synaptic sites [92]. Neuronal exosomes produced following neuronal activity were also taken up by astrocytes where GLT1 was up-regulated as a result, potentially via the indirect action of exosomal miR-124a [31]. Furthermore, neuronal miR-132 packaged into exosomes was found to be internalized by endothelial cells where it altered gene expression and regulated brain vascular integrity [35]. Taken together, these studies point towards a role of extracellular microRNAs in neuronal activity as well as brain development and maintenance.

Glial cells have also been shown to secrete exosomes. Exosomes derived from oligodendrocytes had neuroprotective effects when transferred to neurons and increased neuronal firing rates [55,124]. Schwann cell-derived exosomes altered growth cone morphology of dorsal root ganglion cells [125]. Microglial EV application to hippocampal neurons increased the frequency and the decay of miniature excitatory postsynaptic currents; however, these effects may be independent of EV cargo [126]. Extracellular vesicles of unknown origin, carrying proteins and microRNAs, isolated from rat and human embryonic CSF were found to promote proliferation of neural stem cells in culture [127]. Thus, the literature suggests that non-neuronal cells in the nervous system may utilize exosomes to support neurons and modulate their development and activity. However, the role of microRNAs in these processes is yet to be explored.

### Role in nervous system disease

It is clear that microRNAs contribute to a range of physiological processes in the nervous system by fine-tuning gene expression. Perturbation of this system is likely to lead to disease. Whilst the study of extracellular microRNA function in nervous system disease is still in its infancy, a few extracellular microRNAs have been identified that functionally contribute to disease aetiology (Table 1).

#### Cancer

Due to their diverse roles in a range of critical cellular processes, such as DNA damage response, cell cycle regulation and apoptosis, the role of microRNAs in cancer has been a large focus of microRNA research in recent years. MicroRNAs are thought to be involved in tumourigenesis and tumour progression, by acting as pro-oncogenic oncomiRs or tumour suppressors (reviewed in [128]). Not surprisingly, understanding the role extracellular microRNAs in cancer aetiology and development is of great interest.

Studies suggest that extracellular microRNAs may modulate the tumour environment to favour tumour growth and invasion [129–132]. Exosomes derived from brain-metastatic cancer cells had different microRNA profiles compared with those isolated from cancer cells that were non-brain metastatic. When non-metastatic cells were exposed to exosomes from brain-metastatic cells, they showed greater potential to adhere to brain endothelial cells, suggesting that exosomal microRNA composition may play a role in modulating the metastatic potential of cancer cells [129]. miR-122, which was released in EVs from breast cancer cells, reduced glucose consumption in the brain within astrocytes, thereby promoting brain metastasis [130]. Exosomal miR-19a was released from astrocytes and down-regulated tumour suppressor gene phosphatase and tensin homologue (*PTEN*) in brain metastases, which increased tumour outgrowth [131]. Glioma-derived EVs containing amongst other cargo, cancer-related microRNAs (oncomiRs) such as miR-451 and miR-21, were taken up by microglia, leading to altered cytokine levels which promote glioblastoma invasion and decrease the immune response [132]. Glioblastoma-derived exosomes were also taken up by endothelial cells where they altered gene expression and promoted angiogenesis [133,134]. Tumour-suppressive microRNAs, such as miR-1, were also detected in EVs released from glioblastoma cells [135].

#### Neurodegenerative disease

Cellular microRNAs have been implicated in neurodegeneration in a number of ways. MicroRNAs regulate genes that are involved in the production or clearance of pathological structures [136–139]. This includes genes involved in the production or modification of β-amyloid or tau, which form amyloid plaques and neurofibrillary tangles seen in Alzheimer’s disease brains [136] as well as genes involved in regulating the expression and aberrant accumulation of α-synuclein, a pathological feature of Parkinson’s disease [137,138]. Several microRNAs have been identified that altered expression of the huntingtin gene, thereby regulating huntingtin aggregation in Huntington’s disease [139]. Neurodegenerative disorders are characterized by a progressive spreading of this neuropathology. This ‘spreading effect’ is in keeping with a potential role of EVs in these diseases. Proteins known to be crucially involved in neurodegenerative diseases, such as Aβ and tau in Alzheimer’s disease or prion protein in prion diseases, have been detected in EVs from neurons and glial cells, the uptake of which propagated the cell degeneration and associated cognitive effects [140]. So far, studies have mainly focused on EVs containing disease-related proteins and little is known about the potential role of extracellular microRNAs. Symptoms of Parkinson’s disease can be caused by exposure to neurotoxins, such as 6-hydroxydopamine (6-OHDA). miR-34a within shedding vesicles secreted from LPS-stimulated astrocytes may promote apoptosis in neuronal cells that have been exposed to neurotoxins. Moreover, down-regulation of extracellular miR-34a was able to delay (but not prevent) disease onset in 6-OHDA injected rats [32]. Pro-inflammatory microRNAs have been shown to be up-regulated in brain tissue extracellular fluid and CSF from Alzheimer’s disease patients [141,142]. Exosomes derived from prion-infected neurons contained altered levels of certain microRNAs compared with exosomes from non-infected cells; however their potential functional role was not explored [143].

#### HIV-associated neural disorders

Extracellular microRNAs have also been implicated in HIV-associated neural disorders, caused by the spread of the HIV infection into the brain. Exosomes isolated from infected brain or from infected astrocytes and containing miR-29b or miR-21 could be taken up by neurons and caused neuronal cell death, potentially via necroptosis [144,145].

### Potential treatments

Exosomes in particular are being explored as potential treatment options, especially as an alternative to cell transplantation. Exosomes carrying miR-133b have been isolated from mesenchymal stem cells and have been shown to produce beneficial effects on brain tissue following stroke or traumatic brain injury, including promoting neurite remodelling, angiogenesis and anti-inflammatory effects as well as improving recovery of sensorimotor function and spatial learning [146–149]. In the peripheral nervous system, exosomes released by Schwann cells can be taken up by dorsal root ganglion cells and support axonal regeneration following peripheral nerve injury [125]. Exosomes can also be loaded with artificial small inhibiting RNAs or with exogenously expressed microRNAs known to down-regulate relevant gene targets [37,150,151]. Exosomes carrying tumour suppressor microRNA let-7a have been produced that target epidermal growth factor receptor (EGFR)-expressing breast cancer cells following systemic injection and deliver their functional cargo [150]. With an increasing understanding of the characteristics and cell targeting mechanisms of exosomal vesicles, it may be possible to design exogenous exosomes that selectively target different types of cells [152]. Targeting the brain has additional challenges; however exosomes have been shown to be able to cross the blood–brain barrier [151,153] and may therefore also provide an opportunity to treat CNS diseases.

### Communication between the brain and other tissues

As the importance of communication between the brain and other tissues, such as heart or gut, is becoming more recognized, it has been queried if extracellular microRNAs may play a role in this interaction. Could microRNAs from outside the nervous system shape development, function or health of the brain? As described above, exosomal microRNAs from tumour cells in other parts of the body can reach the brain and prepare the metastatic niche for metastasizing tumour cells [129,130]. Extracellular microRNAs from outside the brain may also play a role in other disease processes. In mice, systemic inflammation was shown to increase EVs and specific microRNAs within the EVs in the CSF. These EVs were observed to cross into the brain parenchyma where they were taken up by astrocytes and microglia and caused pro-inflammatory effects [154]. In contrast, exosomes isolated from various blood cells were able to protect slice cultures from astrogliosis following lipopolysaccharide exposure [155]. Exosomes carrying miR-219 isolated from peripheral blood mononuclear cells may play a role in the improvement of CNS myelination in response to environmental enrichment [155]. In a model of hypoxia during pregnancy, we found that conditioned medium from placental tissue, which contained altered levels of microRNAs, may play a role in modulating neurodevelopment. Application of this placenta-conditioned medium to cortical cultures produced similar neuropathological effects to those seen in the foetal brains of these hypoxic pregnancies. Furthermore, significant correlation between the microRNAs secreted from the placenta and gene expression changes in the foetal brains was observed [156]. Whether microRNAs released from the placenta could modulate foetal brain development under adverse conditions and in normal development by passing into the brain remains to be further explored.

There has also been some evidence that microRNAs from the nervous system may communicate with other tissues, in particular with the heart. A recent study suggested that following cerebral ischaemic stroke, brain endothelial cells release exosomes carrying reduced levels of miR-126 that are taken up by cardiomyocytes, leading to increased mRNA levels of miR-126 target and cardiac dysfunction. The authors suggested that brain endothelium-derived miR-126 may be required for cardiac health after stroke [157].

## Conclusion

More work is needed to fully characterize and elucidate the role of extracellular microRNAs in the nervous system. Much of the work has focused on exosomes but the functional role of microRNAs within these exosomes or within other vehicles was not always investigated. Many of the studies of the molecular mechanisms by which microRNAs may be transferred between cells were performed on tissues other than the nervous system. Although it is likely that some of the mechanisms are universal, this needs to be confirmed. Further characterization of exosomal marker proteins will provide a better understanding why certain EVs are taken up only by specific cell types. As much of the research to date has been performed *in vitro*, it is important to investigate how these identified functions and mechanisms can be translated *in vivo*. The study of extracellular microRNAs is not without its difficulties [158]. While ultracentrifugation is the most common approach to isolating exosomes, it has its caveats due to the potential negative effects on exosome integrity and the requirement for large volumes of starting material. Apart from AGO2 little work has been done on other potential carriers of extracellular microRNAs. There still is a clear need for better methods to be developed for isolation, characterization and analysis of extracellular microRNAs.

Despite these drawbacks, increasing evidence suggests that extracellular microRNAs are not simply the product of apoptotic or necrotic cells nor are they solely the result of the cell discarding unwanted material. Instead these microRNAs are specifically secreted from the cell through active mechanisms and internalized by recipient cells, where they are able to exert their repressive function. These findings support a functional role for extracellular microRNAs in intercellular communication. In the nervous system, extracellular microRNAs may provide a further layer of complexity beyond synaptic transmission by fine-tuning the neuronal network and allow supporting glial cells to modulate the neuronal environment. The potential for communication between the brain and other tissues is particularly intriguing. Lastly, the development of designer exosomes that carry specific microRNAs, cross the blood–brain barrier and target individual cell types could represent a promising advance in drug development.

## Abbreviations

6-OHDA: 6-hydroxydopamine
AGO2: argonaute 2
ALIX: ALG 2-interacting protein X
ATP: adenosine triphosphate
CNS: central nervous system
CSF: cerebrospinal fluid
EGFR: epidermal growth factor receptor
ESCRT: endosomal sorting complex required for transport
EV: extracellular vesicle
HDL: high-density lipoprotein
hnRNPA2/B1: heterogeneous nuclear ribonucleoprotein A2/B1
HuR: human antigen R
LDCV: large dense-core vesicle
MVB: Multivesicular body
NRP1: neuropilin 1
nSMase2: Neutral sphingomyelinase 2
oncomiR: Oncogenic microRNA
P body: Processing body
PTEN: Phosphatase and tensin homologue
RISC: RNA-induced silencing complex
TRBP: TAR RNA binding protein
UTR: Untranslated region
YBX1: Y-box binding protein 1
